# Topography and distribution of adenosine A_2A_ and dopamine D_2_ receptors in the human Subthalamic Nucleus

**DOI:** 10.3389/fnins.2022.945574

**Published:** 2022-08-09

**Authors:** Aron Emmi, Angelo Antonini, Michele Sandre, Andrea Baldo, Martina Contran, Veronica Macchi, Diego Guidolin, Andrea Porzionato, Raffaele De Caro

**Affiliations:** ^1^Department of Neurosciences, Institute of Human Anatomy, University of Padova, Padua, Italy; ^2^Center for Neurodegenerative Disease Research (CESNE), University of Padova, Padua, Italy; ^3^Movement Disorders Unit, Neurology Clinic, University Hospital of Padova, Padua, Italy

**Keywords:** D_2_R, A_2A_R, subthalamic nucleus, receptor-receptor interactions, neuroanatomy, Parkinson’s disease, deep brain stimulation, neurodegeneration

## Abstract

The human Subthalamic Nucleus (STh) is a diencephalic lens-shaped structure located ventrally to the thalamus and functionally implicated in the basal ganglia circuits. Despite recent efforts to characterize the neurochemical and functional anatomy of the STh, little to no information is available concerning the expression and distribution of receptors belonging to the dopaminergic and purinergic system in the human STh. Both systems are consistently implicated in basal ganglia physiology and pathology, especially in Parkinson’s Disease, and represent important targets for the pharmacological treatment of movement disorders. Here, we investigate the topography and distribution of A_2A_ adenosine and D_2_ dopamine receptors in the human basal ganglia and subthalamic nucleus. Our findings indicate a peculiar topographical distribution of the two receptors throughout the subthalamic nucleus, while colocalization between the receptors opens the possibility for the presence of A_2A_R- D_2_R heterodimers within the dorsal and medial aspects of the structure. However, further investigation is required to confirm these findings.

## Introduction

The human Subthalamic Nucleus (STh) is a diencephalic lens-shaped structure located ventrally to the thalamus and functionally implicated in the basal ganglia circuits. While we have previously described the structure, topography and connectivity of the subthalamic nucleus in humans and non-human primates (Emmi et al., 2020), recent work by Alkemade et al. (2019) has focused on the characterization of the functional microscopic anatomy of the structure, with particular regard to the distribution of GABAergic, glutammatergic, dopaminergic and serotoninergic signaling markers. However, despite recent efforts to characterize the neurochemical and functional anatomy of the STh, little to no information is available concerning the expression and distribution of receptors belonging to the dopaminergic and purinergic system in the human STh. Both systems are consistently implicated in basal ganglia physiology and pathology in several movement disorder incuding Parkinson’s Disease (PD), and represent important targets for the pharmacological treatment of motor symptoms in PD. For this purpose, the characterization of dopaminergic and purinergic receptor expression and distribution within the human basal ganglia, and in particular the STh, appears to be highly relevant for their supposed role in the pathophysiology of PD, as previously demonstrated in rodent and non-human primate models.

### Dopaminergic system and receptors

Despite the known connections between the STh and the substantia nigra pars compacta (SNpc) (Emmi et al., 2020) SNpc dopaminergic fibers have been reported to travel across the STh without forming synapses, projecting mainly the striatum (Hedreen, 1999; Alkemade et al., 2015). Only a low number of SN fibers actually appear to form dopaminergic synapses within the STh, mainly in its mediodorsal aspect (Emmi et al., 2020). In rodents, dopaminergic receptor D1 showed variable immunoreactivity that ranged from absent to moderate (Dawson et al., 1986; Dubois et al., 1986; Fremeau et al., 1991; Johnson et al., 1994). Only Savasta et al. (1986) reported a high expression of D_1_R whithin rodent STh, while Mansour et al. (1992) evidenced dense D_1_ receptor binding but no evidence of D_1_ mRNA. D_2_R expression in rodent STh was reported as low by Dubois et al. (1986) and moderate by Johnson et al. (1994). In non-human primates, D_1_ and D_2_ receptors were found presynaptically, on preterminal axons and putative glutamatergic and GABAergic terminals (Galvan et al., 2014). Studies of the human STh displayed no evidence for D_1_R expression (Augood et al., 2000; Hurd et al., 2001) and reported conflicting results as far as D_2_R is concerned, ranging from negative (Augood et al., 2000), to low (Hurd et al., 2001) or moderate (Wang et al., 2001). The expression of other dopaminergic receptors, such as D_3_R and D_4_R, was documented by Wang et al. (2001) and Matsumoto et al. (2002) respectively.

### Purinergic system and receptors

Very little evidence is available on the expression and distribution of purinergic receptors in the human and non-human primate basal ganglia, despite the recent interest in the purinergic modulation of basal ganglia circuitry and the approval of the first purinergic drug for the treatment of Parkinson’s Disease in the United States and Japan. The expression of adenosine receptor A_1_ within the human STh was described by Misgeld et al. (2007), while we are not aware of any studies addressing the presence of A_2_ receptor within the STh in humans.

In the present study, we investigate the distribution of A_2*A*_R purinergic and D_2_ dopaminergic receptors within the rostro-caudal extent of the human STh and the surrounding basal ganglia district. Both receptors have been functionally implicated in the modulation of basal ganglia circuitry and represent potential targets for the pharmacological treatment of movement disorders, such as Parkinson’s Disease, with little-to-no studies addressing their expression and distribution in the human basal ganglia.

## Materials and methods

### Tissue preparation

Ten (10) Human Brains with no history of neurological or psychiatric disorders obtained from the Body Donation Program of the Institute of Human Anatomy of University of Padova (Porzionato et al., 2012; Emmi et al., 2021a,b) were employed for the study. All procedures were carried out according to the ethical standards of the Body Donation Program and to the Declaration of Helsinki. The mean age of the body donors was 56,5 ± 20 years (Table 1). The post-mortem delay was within 24-72h. All specimens were sampled and fixed in-toto in 4% phosphate-buffered formalin solution. Whole-volume coronally sectioned tissue blocks containing the rostro-caudal extent of the STh and the surrounding basal ganglia district were manually processed for paraffin embedding. Paraffin-embedded samples were serially sectioned at 5-micron thickness using a calibrated sliding microtome. Specimen tissue quality and general histopathology was evaluated on Haematoxylin & Eosin stained sections (Porzionato et al., 2020). A fraction of 1/30 serial slides, for an average of 20 ± 2 slides per subject, were employed for immunoperoxidase and immunofluorescent staining.

**TABLE 1:** Clinical data of the study cohort.

Id	Age	Sex	Cause of death
#1	75	M	Myocardial infarction
#2	48	F	Cervical carcinoma, Cardio-respiratory failure
#3	38	M	Carotid artery dissection
#4	36	F	Cardio-respiratory failure
#5	60	M	Cardio-respiratory failure
#6	20	F	Acute Myeloid Leukemia
#7	66	M	Prostatic Cancer
#8	75	F	Cardio-respiratory failure
#9	78	F	Myocardial Infarction
#10	69	M	Cardio-respiratory failure
MEAN	**56,5±20 Years**		

### Immunoperoxidase staining and microscopy

Immunohistochemical staining for A_2A_ Adenosine receptor (7F6-G5-A2, Abcam, Cambridge, United Kingdom, dilution 1:200) and D_2_ Dopamine receptor (Abcam, Cambridge, United Kingdom, dilution 1:100) were employed to characterize receptor distribution within the tissue. Antigen retrieval was performed using DAKO EnVision water bath station; A_2A_R antigen retrieval was performed in a high pH EDTA-buffered bath at 95° Celsius for 15 min, while D_2_R antigen retrieval was performed in a low pH Citrate buffered solution at 95° Celsius for 15 min. Sections were incubated in 0.3 % hydrogen peroxide for 5 min at room temperature to remove endogenous peroxidase activity, and then in blocking serum (3.6% bovine serum albumin A2153, Sigma-Aldrich, Milan, Italy and 0,05% Triton-X in PBS) for 90 min at room temperature. Primary antibodies were incubated for 1h at room temperature followed by correspective secondary antibodies for 30 min at room temperature. Lastly, sections were developed in Diamino-benzidine (DAB, Sigma-Aldrich, Milan, Italy) and counterstained with Haematoxylin. Photomicrographs were acquired with a Leica LMD6 (Leica Microsystems) connected to a Leica DFC320 high-resolution digital camera (Leica Microsystems) and a computer equipped with software for image acquisition (LasX, Leica Microsystems) and analysis (ImageJ). Images of the whole sections were acquired at 20x and 40x magnification and corrected for shading before being loaded into ImageJ software for semi-automatic immunoreactivity quantification, according to previosuly established methodology (Porzionato et al., 2021a,b; Emmi et al., 2022a,b).

### Immunofluorescent staining and microscopy

Fluorescent immunohistochemistry was performed manually. Antigen retrieval was performed on de-paraffinized tissue sections using Dako EnVision PTLink station according to manufacturer recommendations. Following antigen retrieval, autofluorescence was quenched with a 50 mM NH_4_Cl solution for 10 min. Sections were treated with permeabilization and blocking solution (15% vol/vol Goat Serum, 2% wt/vol BSA, 0.25% wt/vol gelatin, 0.2% wt/vol glycine in PBS) containing 0.5% Triton X-100 for 90 min before primary antibody incubation. Primary antibodies were diluted as above in blocking solution and incubated at 4°C overnight; additional immunofluorescent staining was performed for β-III Tubulin (#T8578; 1:300, anti-mouse; #T2200, 1:300, anti-rabbit) and Tyrosine Hydroxylase (#T2928; 1:6000) in combination with the aforementioned A_2A_R and D_2_R antibodies. Alexa-Fluor plus 488 Goat anti-Mouse secondary antibody (Code number: A32723) and Alexa-Fluor plus 568 anti-Rabbit secondary antibody (Code number: A-11011) were diluted 1:200 in blocking solution as above and incubated for 60 min at room temperature. To further avoid background signal and tissue autofluorescence, slides were incubated for 10 min in 0.5% Sudan Black B solution in 70% ethanol at room temperature and abundantly washed with PBS, followed by Hoechst 33258 nuclear staining (Invitrogen, dilution: 1:10000 in PBS) for 10 min. Slides were mounted and coverslipped with Mowiol solution (prepared with Mowiol 4-88 reagent, MerckMillipore, Code number: 475904-100GM). Epifluorescence z-stack images were acquired on a Leica LMD6 Microscope at 20x and 40x magnification. Images were acquired at a 16-bit intensity resolution over 2048 × 2048 pixels. Z-stacks images were converted into digital maximum intensity z-projections, processed, and analyzed using ImageJ software.

### Semi-automatic immunoreactivity quantification

Serial sections of each STh were divided into rostral, central and caudal thirds (6 ± 2 slides per level). Digital photomicrographs of the lateral, dorsal, ventral and medial sectors were taken for each slide and morphometrical values were averaged within sections belonging to each assigned level, according to the schematization seen in Lévesque and Parent (2005), as seen in Figure 4. Digital photomicrographs taken with the aforementioned scheme were loaded as stack in ImageJ. The area of the sections was quantified by manually drawing the boundaries of the specimens. A Maximum Entropy Threshold was applied and manually adjusted for each section in order to discern immunoreactive elements from background and negative tissue. Quality control of the applied threshold was performed by an expert morphologist by overlying the thresholded images to the original photomicrographs. Particle analysis was employed with an experimentally defined pixel threshold in order to evidence immunoreactive elements quantity and total area, expressed as percentage (A%) within the digital image.

**FIGURE 1:**
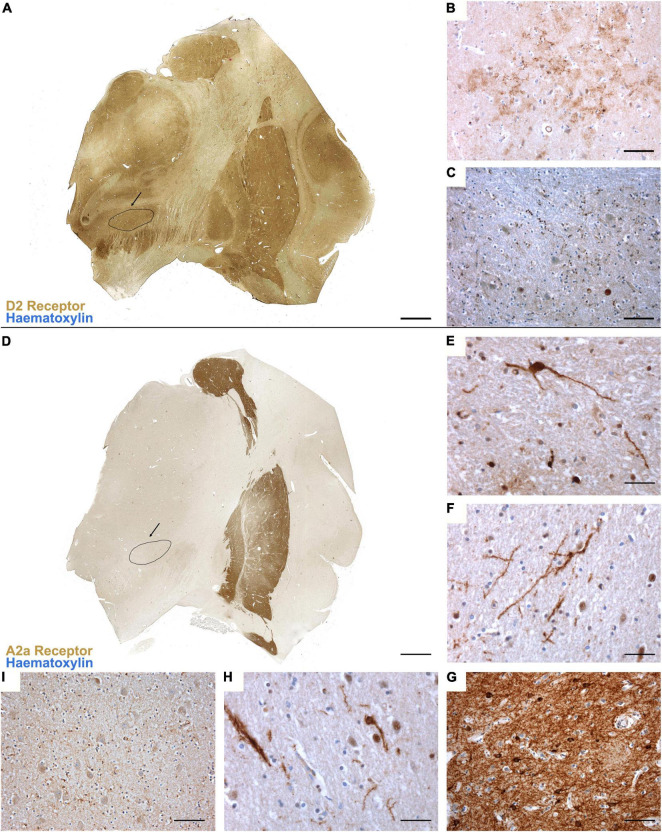
**(A)** Topographical distribution of D_2_ dopamine receptor in the basal ganglia. Immunoperoxidase staining reveals a moderate, yet diffuse, distribution of the receptor, with no marked differences between the striatum, globus pallidus and subthalamic nucleus (evidenced in black). **(B)** D_2_R immunoreactivity in the striatum and **(C)** in the subthalamic nucleus, where mainly neurites and dot-like reactivities are marked. **(D)** Topographical distribution of A_2A_ adenosine receptor in the basal ganglia reveals a marker expression of the receptor in the striatum **(G)** and external globus pallidus, with very mild expression in the internal globus pallidus **(E)**, claustrum **(F)**, thalamus and subthalamic nucleus [evidenced in black, **(H,I)**].

**FIGURE 2:**
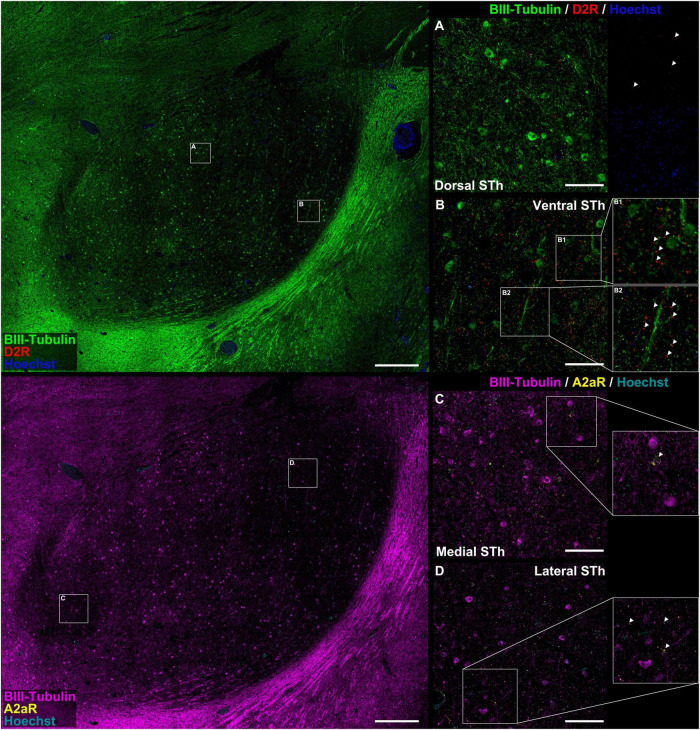
Colocalization study between A_2A_R / D_2_R proteins and pan-neuronal marker β-III-tubulin. There appear to be significant differences in the distribution of D2 receptor immunofluorescent signal between the dorsal **(A)** and ventral **(B)** STh. In both instances, immunoreactivities mostly colocalized with β-III-tubulin neuritic structures at the level of dendritic spines [magnification in **(B)**]. In some occasions, D_2_R did not colocalize with β-III-tubulin, suggesting for the expression of the receptor also in non-neuronal cells. A_2A_R can be found as dot-like immunoreactivities colocalizing with β-III-tubulin positive neurites or as rare somatic cytoplasmic reactivities **(C,D)**, even though non-β-III-tubulin colocalizing reactivities were also found.

**FIGURE 3:**
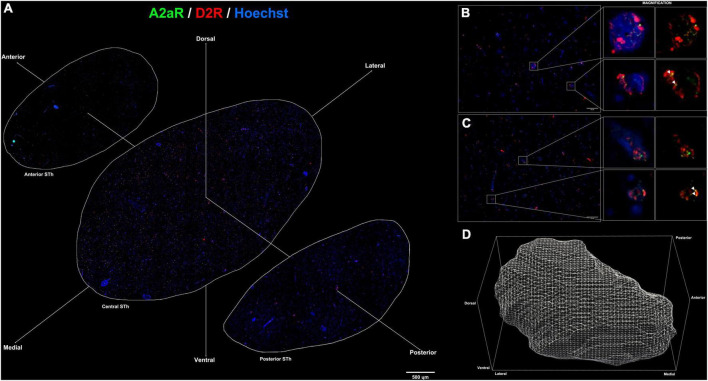
**(A)** Immunofluorescent staining for A_2A_R (green) and D_2_R (red) throughout the rostro-caudal extent of the subthalamic nucleus reveals a peculiar topographical distribution. **(B,C)** colocalization of A_2*A*_R and D_2_R proteins within the subthalamic nucleus (white arrows). **(D)** 3D representation of the structure of the subthalamic nucleus following reconstruction of serial sections.

**FIGURE 4:**
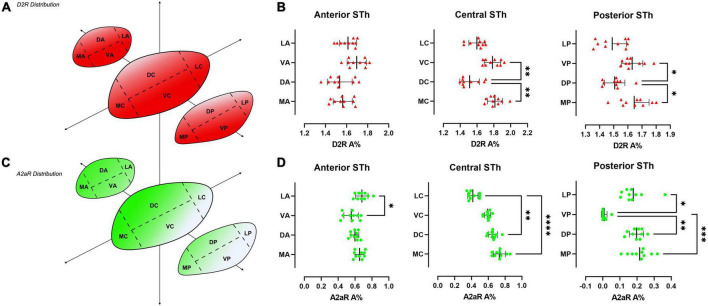
Heatmap of the distribution of D_2_R **(A)** and A_2A_R **(C)** throughout the rostro-caudal extent of the STh, indicating a decreasing ventral-to-dorsal gradient for D_2_R, and an opposite decreasing dorsomedial-to-ventral gradient for A_2A_R. **(B,D)** Non-parametric ANOVA (Friedman’s test) of the different levels of sectioning throughout the STh reveals topographical differences within specific STh subregions.

### Statistical analyses

Statistical Analyses. Statistical analyses and visualizations were performed using GraphPad Prism 9. One-way non-parametric ANOVA (Friedman’s test) was performed to evaluate receptor distribution throughout STh sectors, as seen in Figures 4B,C. Further statistical details for each plot can be found in the corresponding figure legend. Throughout the text * indicates *p* < 0.05, ^**^ *p* < 0.01, ^***^ *p* < 0.001 and ^****^ *p* < 0.0001.

## Results

### Distribution of A_2A_ and D_2_ receptors

Immunoperoxidase staining for A_2A_ adenosine and D_2_ dopamine receptors in the basal ganglia revealed a peculiar topographical organization, as shown in Figure 1. While D_2_R appears to be uniformly distributed with mild-to-moderate expression within the basal ganglia (Figure 1A), A_2A_R immunoreactivity is particularly marked at the level of the Caudate Nucleus, Putamen and External Globus Pallidus (GPe) (Figures 1D,G), with very mild immunoreactivity detectable within the Claustrum (Figure 1F), Thalamus, Subthalamic Nucleus (Figures 1H,I) and Internal Globus Pallidus (Figure 1E). In the STh, D_2_R immunoperoxidase staining revealed a predominantly neuritic immunoreactivity (Figure 1C), with dot-like reactivities representing the most common finding; sporadic cytoplasmic immunoreactivity of neuronal somata was also detected (range: 1–2 somatic reactivities per mm^2^). Similarly, A_2A_R immunoperoxidase staining revealed mild reactivity detectable as oblongated neurite-like structures and sparse dot-like reactivities. Sporadic cytoplasmic immunoreactivity of neuronal somata was also detected (range: 1–2 somatic reactivities per mm^2^).

### Distribution of D_2_ receptors in the human Subthalamic Nucleus

Double immunofluorescent staining for β-III-tubulin, a pan-neuronal marker, and receptor proteins (A_2A_R / D_2_R) confirmed immunoperodidase staining findings (Figure 2). D_2_R reactivity was found predominantly at the level of the dendritic spines of β-III-tubulin positive neurites (Figures 2A,B,B1,B2); while colocalization of D_2_R signal (red channel) with β-III-tubulin (green channel) was predominant, sporadic non-colocalizing D2R signal was detected, compatible with reports in literature describing D_2_R expression in non-neuronal (β-III-tubulin negative) cells, such as astrocytes (Pelassa et al., 2019). D_2_R reactivity appears to follow a ventromedial-to-dorsal decreasing gradient (Figures 2A,B, 3A, 4A). As seen in Figures 3A, 4A, there appears to be a statistically significant difference in D_2_R density between the Ventral and Dorsal STh in both the central (VC vs. DC) and posterior (VP vs. DP) third (*p* = 0.0017; *p* = 0.0194), as well as the Ventral and Medial STh (*p* = 0.0017; *p* = 0.0109); no difference was found between the ventral STh and the lateral pole, and throughout all sectors of the anterior third of the STh (*p* > 0.05).

### Distribution of A_2A_ receptors in the human Subthalamic Nucleus

As seen in Figures 2C,D, A_2A_ receptor seems to be localized with a similar sub-cellular distribution as D2 receptors; indeed, most of the A_2*A*_ signal was detected as dot-like reactivities colocalizing predominantly with β-III-Tubulin positive neurites (Figure 2D), with the exception of sporadic somatic reactivites (Figure 2C) and non-β-III-tubulin positive structures [likely glial cells, as previously reported by Pelassa et al. (2019)]. Topographically, A_2A_R appear to follow a dorsal to ventral decreasing gradient, as seen in Figures 3A, 4C. Single-way Friedman test revealed statistically significant differences in A_2A_R density within these sectors of the STh, as seen in Figure 4D. While the distribution of A_2A_R appears to be more uniform within the anterior STh, marked differences between ventral and dorso-medial / dorso-lateral sectors become evident at the level of the central and posterior STh.

### Colocalization of A_2A_ and D_2_ receptors

Limited to the information obtained by double-immunofluorescence assays, colocalization between A_2A_ and D_2_R signals was found throughout the STh, with a more prominent distribution of colocalizations found at the level of the medial and dorsal STh, as seen in Figures 3A–D. Interestingly, despite the lower distribution of D_2_ receptors in the dorsal STh, the presence of A_2A_R colocalizations within the dorsal STh may indicate the presence of A_2A_- D_2_ receptor heterodimers, which are known to modulate dopaminergic afferences in the striatum. On the other hand, colocalization of D_2_R and A_2A_ immunofluorescent signal does not prove the presence of A_2A_-D2 heterodimers, which needs to be investigated with appropriate methodologies, such as proximity ligation assay (PLA).

## Discussion

A_2*A*_ receptors are known to play an important role within the basal ganglia circuitry, in particular as postsynaptic facilitators in the GABAergic striato-pallidal neurons of the indirect pathway. Furthermore, A_2A_R are also expressed in the presynaptic terminals of glutamatergic neurons, in particular the context of cortico-striatal and thalamo-striatal pathways. In the STh, A_2A_R was detected at the level of β-III Tubulin immunoreactive dendritic spines and also in non-neuronal cells. Our findings also suggest a topography-specific distribution of A_2A_R, particularly in the anterior, medial and dorso-lateral STh. According to the morpho-functional subdivision of the STh, the anterior STh, as well as the medial thirds of the central and rostral STh, are functionally related to the limbic circuit, while the dorso-lateral STh is involved in the motor circuits, suggesting for a more prominent purinergic modulation of the limbic and motor STh when compared to the ventral (associative) STh.

Similarly, D_2_R are known to act as modulators of glutamatergic synapses (Schiffmann et al., 2007). In the STh, we detected D_2_R with a similar sub-cellular distribution as A_2A_R, mainly as reactivities on dendritic spines and on sporadic non-neuronal cells. The topography of D_2_R within the STh indicates a decreasing gradient between the ventral and medial STh toward the dorsolateral STh. This suggests for a more prominent role of D_2_R modulation at the level of the associative (ventral) and limbic (medial) regions of the STh, rather than the motor (dorsal) regions of the STh. Interestingly, Tyrosyne Hydroxylase (TH) staining revealed a conspicuous bundle of cathetcolaminergic fibers originating from the substantia nigra (SN), coursing below the ventral aspect of the STh, and directed laterally toward the Striatum (Supplementary Figure 1, passing through the central STh). Sporadic TH+ fibers were found entering the STh from the ventral aspect, likely as collaterals of the ventrally coursing nigro-striatal fibers, with little-to-no ramifications coursing toward the dorsal aspect of the STh. This is likely associated to the lower density of D_2_R in the dorsal-central and -posterior STh.

The detection of colocalizing A_2A_R and D_2_R in topographically defined regions of the STh represents an intriguing finding that requires further investigation, with particular regard to its implication in the modulation of basal ganglia circuitry in health and disease. *In situ* Proximity Ligation Assay (PLA) (Narváez et al., 2021), as well as co-immunoprecipitation, can be employed to further confirm double-immunofluorescence findings. Interestingly, colocalization between the two receptors appears to be most prominent within the medial and dorsolateral STh. A_2A_ and D_2_ receptors are often co-expressed in glutamatergic terminals, where they interact by heterodimerization with an antagonistic relationship, as the activation of a receptor inhibits the action of the other in controlling glutamate release (Guidolin et al., 2020). The antagonistic relationship between A_2A_R and D_2_R leads to reduced D_2_R recognition and G_*i/0*_ coupling (Guidolin et al., 2020) and, consequently, an inhibition of the Ca^2+^ influx over the L-type voltage dependent calcium channels, thus modulating the excitability of these neurons (Fuxe et al., 2010). The detection of colocalizing A_2A_R and D_2_R, particularly in the dorsal STh, may indicate a selective role of receptor heteromers in modulating neuronal activity in the motor regions of the STh.

Given their role in the regulation of motor function within the Basal Ganglia, A_2A_ and D_2_ receptors were evaluated as a Parkinson’s Disease therapeutic target, basing on the results obtained with co-administration of A_2A_ receptor antagonist and L-DOPA or preferential D_2_R antagonists (Ferré et al., 1997). The involvement of A_2A_R - D_2_R heteromers in the pathogenesis of PD appears to be supported by the higher level of expression of heteromers in the Striatum of PD patients (Fernández-Dueñas et al., 2019). In fact, the antagonistic interaction between A_2A_R and D_2_R appears to be increased in the striatum of murine models of Parkinson’s disease (Ferré and Fuxe, 1992; Schwarzschild et al., 2006; Fuxe et al., 2010). According to Schiffmann et al. (2007), A_2A_ and D_2_R receptors are expressed in striatal GABAergic neurons involved in motor functions with a facilitator and inhibitory role, respectively. Furthermore, the expression of D_2_R, A_2A_R and mGluR5 heteromers, and therefore the interaction between these receptors, within the dendritic spines of dorsal and ventral striato-pallidal GABAergic neurons involved in PD, may suggest an involvement of these receptors in the pathogenesis of PD (Fuxe et al., 2003). The role of A_2A_R antagonists has been evaluated in several rodent PD models, in which it dose-dependently increases motor activity, when combined with L-DOPA and D_2_R agonists at sub-threshold doses; Also, Parkinson-related motor symptoms have been consistently treated in rodents and non-human primates following administration of A_2A_R antagonists (Ferré and Fuxe, 1992; Tanganelli et al., 2004; Schwarzschild et al., 2006; Fuxe et al., 2007). Fuxe et al. (2010) hypothesize that A_2A_R antagonists, targeting A_2A_R- D_2_R heteromers, increase D_2_R signaling. The administration of these antagonists should not have an effect on the expression of heteromers, as they have been found to be constitutive (Canals et al., 2003) and, therefore, present in the absence of their agonists. In the context of PD treatment, the enhancement of A_2A_R - D_2_R heteromers internalization induced by L-DOPA administration causes a compensatory up-regulation of A_2A_R homomers (Fuxe et al., 2010), which induces an increase in protein phosphorylation including ion channels. This mechanism could eventually stabilize pathological receptors mosaics that tend to form within the transcriptional signaling induced by D_2_R excessive activation caused by L-DOPA treatment (Ciruela et al., 2004; Woods et al., 2005). This complex reaction-chain leads, in the end, to an impaired firing pattern in the striato-pallidal GABA pathways which is responsible for dyskinesias (Fuxe et al., 2010). To date Istradefylline is the only approved A_2A_R antagonist drug. This drug, when combined with L-DOPA or D_2_R agonists has been proved by several randomized placebo controlled studies to reduce the off-time and improve motor symptoms, while a worsening in L-dopa long-term treatment-induced dyskinesia has been evidenced, although publication bias does not allow to affirm it with certainty (Sako et al., 2017). Intriguing results have been shown by Collins-Praino et al. (2013), who studied the effect of Deep Brain Stimulation (DBS) in a rodent model of Parkinson’s disease which presented dopamine antagonists-induced tremor jaw movements (TJM). DBS induced a significant reduction of TJM, and its effect was dependent on the frequency and intensity of the stimulation. An interesting result was evidenced in a group of rats to which an A_2A_R antagonist drug (MSX-3) was administered along with DBS. A_2A_R antagonist treatment enhanced the tremorolytic effect of DBS, allowing to obtain the same clinical results with a lower intensity and frequency of stimulation. Previous studies already proved the synergistic effect of L-DOPA treatment on DBS (Bejjani et al., 2000), but A_2A_R antagonists may constitute a complementary strategy to prevent levodopa-induced motor complications (Collins-Praino et al., 2013).

## Conclusion

Our study describes for the first time the topography and the distribution of A_2A_ adenosine and D_2_ dopamine receptors in the human STh. Our findings indicate a peculiar distribution of these receptors throughout the STh, with dopamine D_2_R presenting a more diffuse distribution with a decreasing ventral to dorsal gradient, and A_2A_R presenting a more mild distribution with a distinct dorsal to ventral decreasing gradient. The identification of colocalizations between A_2A_ adenosine and D_2_ dopamine receptors in the medial and dorsal STh suggests the presence of receptor-receptor interactions in the form of A_2A_R -D_2_R heteromers.

## Data availability statement

The raw data supporting the conclusions of this article will be made available by the authors, without undue reservation.

## Ethics statement

Ethical review and approval was not required for the study on human participants in accordance with the local legislation and institutional requirements. The patients/participants provided their written informed consent to participate in this study.

## Author contributions

AA, AP, and AE designed the study. RD, AP, and AE sampled the brains. AE and MS performed immunohistochemical staining and the morphometrical evaluation. AE performed the statistical analyses and drafted the figures. AE, AP, DG, AA, and MS drafted the manuscript. All authors reviewed and approved the final version of the manuscript.

## Supplementary material

Supplementary Figure 1 Tyrosine hydroxylase (TH) (green) immunofluorescent staining of the subthalamic district reveals a distinct TH+ bundle of axon coursing ventrally to the STh.
