# Supplementary figures and images for: Topography and distribution of adenosine A2A and dopamine D2 receptors in the human Subthalamic Nucleus
Source: Front Neurosci. 2022 Aug 9;16:945574. doi: 10.3389/fnins.2022.945574 (PMC9396224; doi:10.3389/fnins.2022.945574)

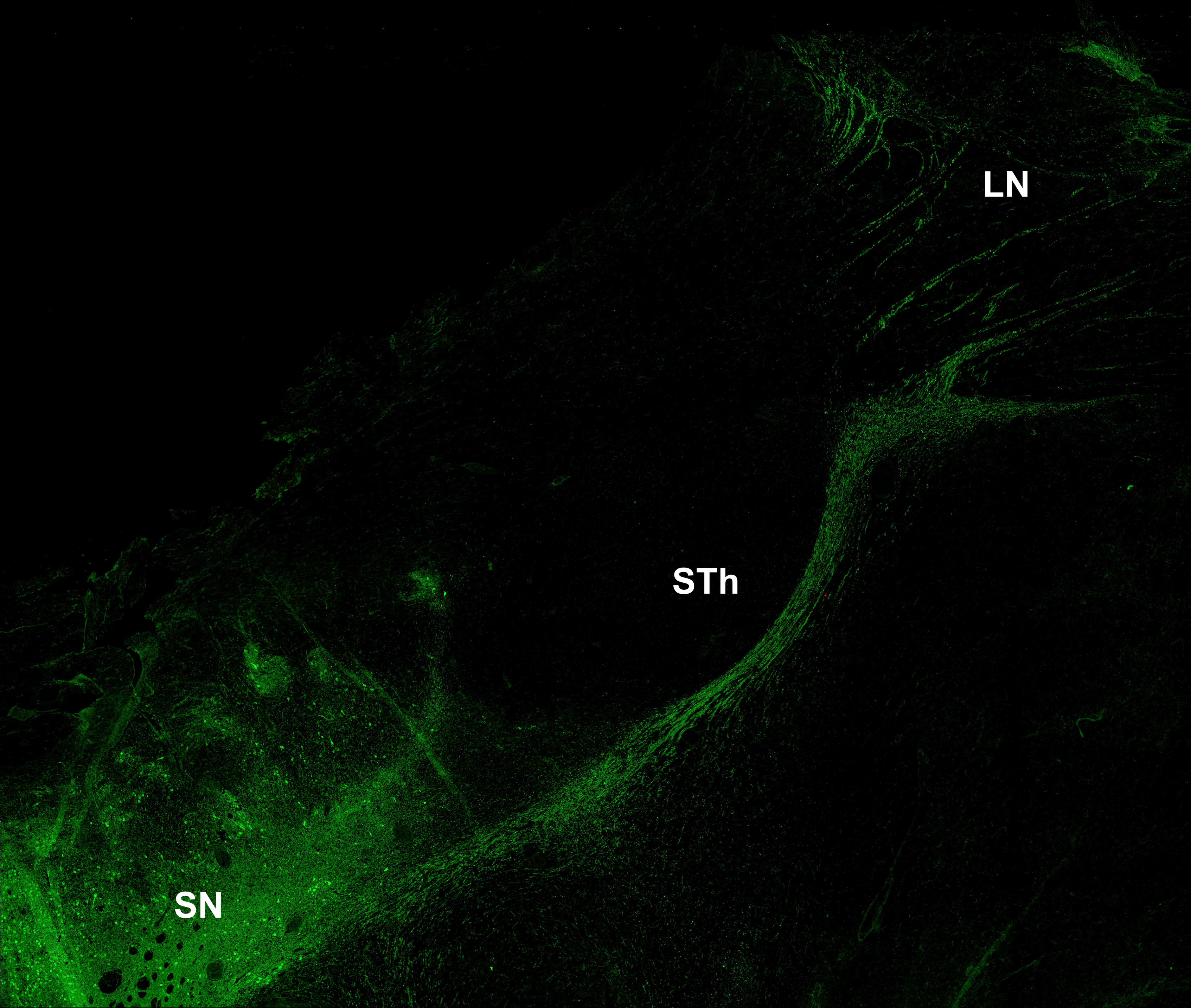

Supplement: Supplementary Figure 1 — Tyrosine hydroxylase (TH) (green) immunofluorescent staining of the subthalamic district reveals a distinct TH+ bundle of axon coursing ventrally to the STh. [file Image_1.JPEG]
